# Influence of Micropatterned Grill Lines on *Entamoeba histolytica* Trophozoites Morphology and Migration

**DOI:** 10.3389/fcimb.2018.00295

**Published:** 2018-08-24

**Authors:** Francisco Sierra-López, Lidia Baylón-Pacheco, Patricia Espíritu-Gordillo, Anel Lagunes-Guillén, Bibiana Chávez-Munguía, José L. Rosales-Encina

**Affiliations:** Departamento de Infectómica y Patogénesis Molecular, Centro de Investigación y de Estudios Avanzados del Instituto Politécnico Nacional, Mexico City, Mexico

**Keywords:** *Entamoeba histolytica*, micropatterned grill lines, migration, fibronectin, erythrocyte extract, filopodia, pseudopods, lamellipodia

## Abstract

*Entamoeba histolytica*, the causal agent of human amoebiasis, has two morphologically different phases: a resistant cyst and a trophozoite responsible for the invasion of the host tissues such as the colonic mucosa and the intestinal epithelium. During *in vitro* migration, trophozoites usually produce protuberances such as pseudopods and rarely filopodia, structures that have been observed in the interaction of trophozoites with human colonic epithelial tissue. To study the different membrane projections produced by the trophozoites, including pseudopods, filopodia, uropods, blebs, and others, we designed an induction system using erythrocyte extract or fibronectin (FN) in micropatterned grill lines (each micro-line containing multiple micro-portions of FN or erythrocyte extract) on which the trophozoites were placed in culture for migration assays. Using light, confocal, and scanning electron microscopy, we established that *E. histolytica* trophozoites frequently produce short and long filopodia, large retractile uropods in the rear, pseudopods, blebs, and others structures, also showing continuous migration periods. The present study provides a simple migration method to induce trophozoites to generate abundant membrane protrusion structures that are rarely obtained in normal or induced cultures, such as long filopodia; this method will allow a–better understanding of the interactions of trophozoites with FN and cell debris. *E. histolytica* trophozoites motility plays an important role in invasive amoebiasis. It has been proposed that both physical forces and chemical signals are involved in the trophozoite motility and migration. However, the *in vivo* molecules that drive the chemotactic migration remain to be determined. We propose the present assay to study host molecules that guide chemotactic behavior because the method is highly reproducible, and a live image of cell movement and migration can be quantified.

## Introduction

*Entamoeba histolytica*, the causative agent of human amoebiasis, presents in its life cycle **two** stages, trophozoite and cyst (WHO, 1997; Ali et al., 2012). Trophozoites correspond to the motile and invasive form of the parasite that usually resides in the human large intestine, and occasionally penetrate intestinal mucosa and migrate to other organs such as the liver, lungs or brain (Petri and Haque, 2013). During intestinal infection, trophozoites disrupt, and degrade intestinal mucosa barrier, and can also trogocyte mucosal epithelial cells and promote cell death by multiple cytotoxic mechanisms (Ralston et al., 2014; Begum et al., 2015). In the course of invasion, trophozoites interact with extracellular matrix components (ECM) such as fibronectin (FN), laminin and collagen (Solaymani-Mohammadi and Petri, 2008), which *in vitro* induce actin cytoskeleton remodeling that is involved in adhesion, migration (Sengupta et al., 2009; Javier-Reyna et al., 2012) and motility (Aguilar-Rojas et al., 2016).

Migration, associated with changes in trophozoite morphology, is stimulated by different factors such as the need for nutrients or the chemoattractant environment, which induces the characteristic amoeboid movement of trophozoites (Aguilar-Rojas et al., 2016). In two-dimensional cell culture surfaces, the migration, and motility of trophozoite usually show a fast generation of blebs and pseudopodia protrusions at the leading edge (Maugis et al., 2010). Occasionally retracting uropod at the rear end and short or large filopodia are produced by trophozoites in normal culture (González-Robles and Martínez-Palomo, 1983; Marquay Markiewicz et al., 2011), therefore these structures are generally not mentioned in the description of the mobility of the parasite (Aguilar-Rojas et al., 2016). However, filopodia of 1-6 micrometers extending between the trophozoites and MDKC or Caco-2 cell monolayers were reported (Li et al., 1994). In addition, trophozoites in contact with the mucus and epithelial cells, in an “*ex-vivo* human intestinal model,” show short filopodia (Bansal et al., 2009).

FN-coated surfaces induce a wide variety of cellular responses in trophozoites, such as focal binding, degradation *in situ* of FN, remodeling of actin and myosin cytoskeleton, adhesion structures formation (Meza, 2000; Emmanuel et al., 2015), and pseudopodia and lamellipodia formation (Talamás-Lara et al., 2015). Some stress or toxic conditions of culture such as the treatment of trophozoites with α-linoleic acid causes the formation of large filopodia (about 5 μm) and loss of directional motility followed by cell death of the trophozoites (Manna et al., 2013).

Lysed red blood cells (RBCs), bacteria, ECM proteins, and TNF represent chemotactic stimulus in trophozoites (Zaki et al., 2006). Chemoattractant components such as ECM proteins have been used as substrate during *in vitro* adhesion and migration assays, methods that enable the study of confined cell migration (Paul et al., 2017), such as microcontact-printed and micro patterns of substrate (micropatterns symmetric or asymmetric) on different surfaces to study both cell morphology and protein expression (Jiang et al., 2005; Alamdari et al., 2013; Kim et al., 2013; Paul et al., 2016). Methods for micropatterning cells culture usually require a complex and specialized equipment that is not readily accessible in most laboratories but other simple and fast methods to obtain micropatterns have been performed, such as the “Parafilm^TM^ insertion method” (plated cells into circular or striped micropatterns) used to culture ARPE-19 and MDCK epithelial cells (Javaherian et al., 2011). Actually, micropatterns of a substrate such as continuous micropattern lines have been used to study the morphology of cancer cells during migration. These micropatterns allow an efficient formation of different large membrane protrusion, directional migration, and identification of crucial proteins related to cellular mobility (Théry, 2010; Paul et al., 2016; Tocco et al., 2018), and even facilitate the characteristic amoeboid cell migration which frequently shows pseudopodia, uropods, lamellipodia and filopodia structures in these abnormal cells (Théry, 2010; Fruleux and Hawkins, 2016; Paul et al., 2017).

Here we present a method that uses glass or plastic surfaces covered with a substrate in a “micropatterned grill line” (MPGL), which spatially stimulate *E. histolytica* trophozoite adhesion, migration, and an efficient formation of different membrane protrusions.

## Materials and methods

### Human samples for migration assays

Human blood was obtained from voluntary donors to purify fibronectin from plasma or erythrocytes; these materials were used to prepare Micropatterned Grill Lines for migration assays with trophozoites from *Entamoeba histolytica*. The procedure for obtaining fresh blood from volunteers was carried out under the international guidelines established for the study in human populations of the Declaration of Helsinki.

### *E. histolytica* culture

*E. histolytica* trophozoites, strain HM1:IMSS (ATCC 30459), clone A (Ramírez-Tapia et al., 2015), were axenically cultured at 37°C in TYI-S-33 medium supplemented with 10% heat-inactivated adult bovine serum (ABS) and harvested during logarithmic growth phase (Diamond et al., 1978). All experiments presented here were performed on at least three separate occasions and in triplicate.

### Fibronectin purification

FN was purified by the gelatin-sepharose affinity chromatography method (Ruoslahti et al., 1982) from fresh human blood collected in 5% sodium citrate and 10 mM phenylmethylsulfonyl fluoride (PMSF). Protein purity was monitored by SDS-PAGE. Affinity purified FN was dialyzed against 0.15 M NaCl, 0.05 M Tris-HCl (pH 7.4), and stored at −70°C. FN was quantified using the extinction coefficient of 1.28 ml/mg-cm at 280 nm and was suspended to a final concentration of 0.2 μg/μl.

### Red blood cells extract preparation

Human blood (40–80 μl) was obtained by finger prick and transferred into 1 ml of PBS. Red blood cells (RBC) were washed three times with PBS (320 x *g*) and diluted to a final concentration of 1000 RBC/μl in PBS. RBC (10 ml) were sonicated at 90% amplitude for 8–10 s (Ultrasonic processor GE 100), and the obtained extracts used immediately.

### Micropatterned grill lines (MPGLs) preparation

FN (1–2 μl, 0.2 μg/μl) or RBCs (1,000 RBCs/μl) were placed on glass or plastic surfaces and subsequently spread over an area of about 2 × 15 mm. From this area, several thin lines were extended that were dried with a continuous air flux and sterilized under UV for 5 min. Substrate excess in the 2 × 15 mm dry fringes was removed by scraping and this area was used as a site to seed cells. For motility assay by light microscopy, pieces of Parafilm “M” (Bemis Flexible Packaging, Neenah, WI 54856) were placed on each long side of the glass slide and the coverslips. The separation (depth) between glass slides and coverslips were 0.1–0.2 mm. Plastic barriers were placed on the periphery of both glass slider sides without a coverslip. The glass slider periphery in contact with Parafilm, coverslip, and plastic was sealed with nail varnish (Supplementary Figure 1) (MPGLs chamber).

### Light microscopy of living cells

Medium TYI-S-33 was placed in the space between the coverslip and glass slider, then 2 × 10^5^ trophozoites in 150–200 μl of TYI-S-33 medium were gently seeded on the start site of the MPGLs (dry fringe) and incubated for 15–20 min at 37°C. Supernatant from the “seeding area” was removed and 400 μl of TYI-S-33 medium was added to the two sites of the chamber lined with plastic to continue incubation at 37°C for 1–5 h (Supplementary Figure 1B). The MPGLs chambers were incubated at different times, using as reference the 2 h of incubation by light microscopy at 35–37°C.

### Migration assay

For the migration assay, trophozoites plated in culture media on the MPGLs start site, as above, were incubated for 15 min at 37°C (Supplementary Figure 1C). Non-adherent trophozoites were washed out and then adherent trophozoites were preincubated for 2 h in complete medium and thereafter monitored every 20 sec for 2 min by light microscopy.

### Migration rate

The migration rate was determined after 120 min of culturing the trophozoites on the MPGLs (FN or erythrocyte extract) at 37°C. Images were captured from videos at a rate of six frames/min. We applied ImageJ (http://imagej.nih.gov/ij/) to obtain the region of interest (ROI) with the function “adjust ellipse,” and the geometric center and the front of each cell was determined. Cells that had migrated more than 3 mm from the initial front of the seeding in the MPGLs were chosen. The velocity of a mobile cell and the time of migration was determined by plotting the geometric center movement every 10 s. Non-stimulated trophozoites were evaluated as the negative control.

### Scanning electron microscopy (SEMs)

Trophozoites (2 × 10^5^) in 150–200 μl of TYI-S-33 medium were seeded on the start site of the MPGLs (dry fringe) and incubated for 15–20 min at 37°C, then the supernatant was removed and enough TYI-S-33 medium was added to cover the entire surfaces (**Figure 2B**). Trophozoites were incubated at 37°C in a humid chamber for 1–5 h. Trophozoites were fixed with 2.5% (v/v) glutaraldehyde in 0.1 M sodium cacodylate buffer pH 7.2 and dehydrated with increasing concentrations of ethanol. Samples were critically point dried with CO_2_ in a Samdri-780 Tousimis apparatus. Then, they were gold coated in an ion-sputter device (Jeol-JFC-1100) and examined with a Jeol JSM-7100F field emission scanning electron microscope (Chávez-Munguía and Martínez-Palomo, 2011).

### Confocal microscopy

Trophozoites were incubated at 37°C on the MPGLs (FN or RBCs extract as substrate) or uncoated coverslips for 2 h in TYI-S-33 medium and fixed with 4% p-formaldehyde in PBS for 45 min at 37°C; then they were washed with PBS and permeabilized with 0.1% SDS and 0.06% Triton X-100 in PBS for 8 min at room temperature and washed with PBS. F-actin was stained with rhodamine-phalloidin (Molecular Probes, Sigma, 1:200) for 30 min at room temperature. Samples were mounted onto glass slides with VectaShield with DAPI (Vector Laboratories Inc., Burlingame, CA, USA) and observed under a Carl Zeiss LSM 700 confocal microscope.

## Results

### Elaboration of MPGLs

To explore how the MPGLs can regulate *E. histolytica* trophozoites migration, we used RBCs extract or FN as the substrate to construct pattern arrays on glass or plastic surfaces. We fabricate topographic patterns that were composed of parallels arrays with two different volume of substrate solutions respectively (Supplementary Figure 1). The dry lines of the MPGLs were 20–100 μm wide and 1–5 cm long, and the amount of protein was 5 μg/cm^2^ for FN and 1,000 ± 500 μg/cm^2^ for RBCs extract. To ensure binding on glass or plastic surfaces and formation of the micrometric substrate arrays, it is crucial to apply the substrate solution under a continuous air flux. Assay chambers were made by forming a “coverslip-glass slider” sandwich to observe the mobility of the trophozoites stimulated by the MPGLs.

### Incubation of trophozoites on the MPGLs

**Figure 1:**
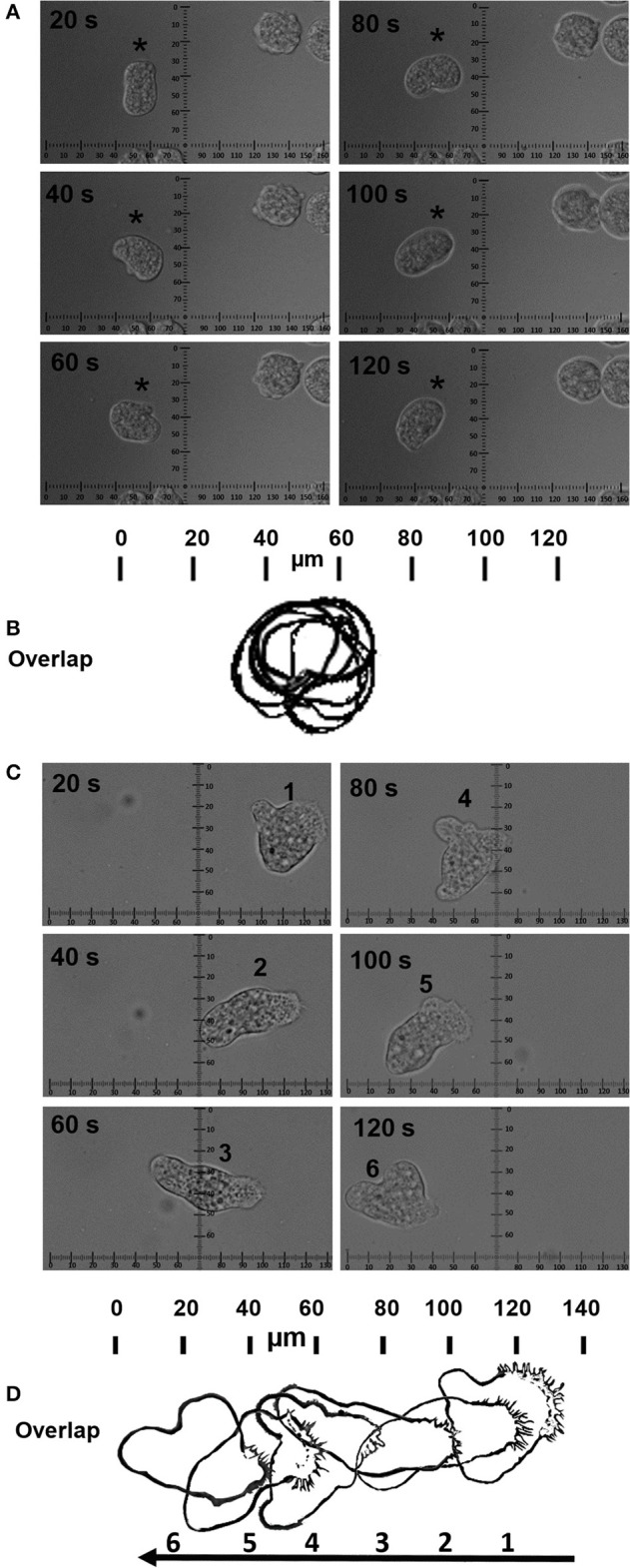
Influence of the MPGLs on *E. histolytica* migration. Time-lapse images (in seconds) of trophozoites incubated without **(A)** or with stimulation (MPGLs) **(C)**. Overlap of the schematic periphery of one trophozoite (asterisk) at the indicated times without **(B)** or with stimulation **(D)**. The arrow indicates the direction of the trophozoite migration. (Scale bar in **(A,C)**: 20 μm; Scale bar in **(B,D)**: 10 μm).

Trophozoites were seeded on the MPGLs start sites and cell morphology was monitored during the cell-substrate interaction. Trophozoites induced by MPGLs showed a response that was significantly different from those non-stimulated trophozoites (Figure 1). Trophozoites in contact with the MPGLs generally showed two phases alternating between them at different times. The first is a phase of non-migration in which they remained on the MPGLs (but showed plasma membrane movement, membrane protrusions as large filopodia), and the second in which trophozoites migrate rapidly on MPGLs (showing the characteristic pseudopod and others membrane protrusions).

**Figure 2:**
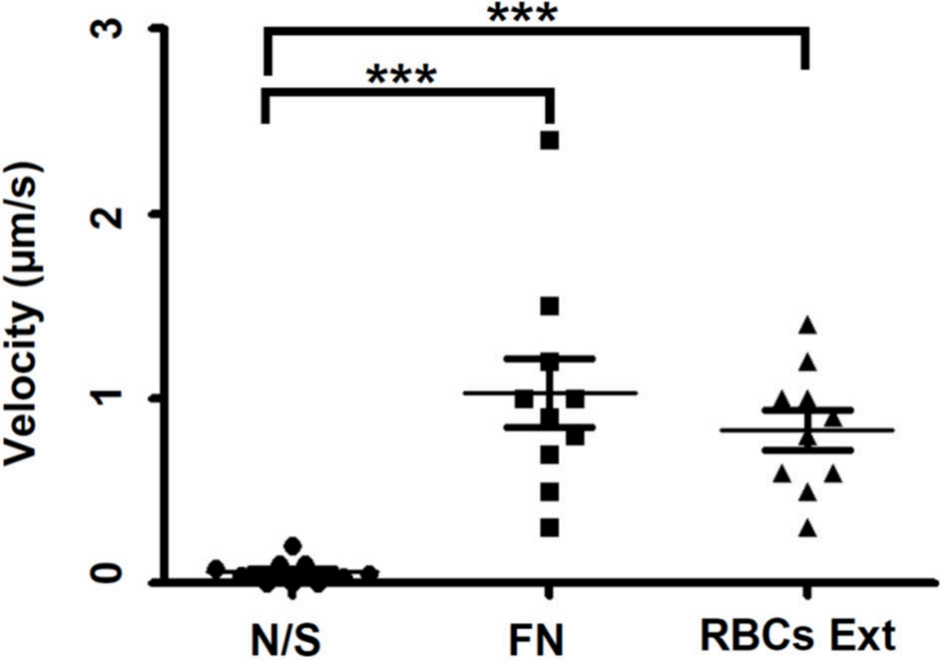
Influence of the MPGLs on the velocity of trophozoites' movement. Velocity of cells movement was determined for individual trophozoite on the MPGLs (FN or RBCs extracts). Three experiments were done for each MPGLs type. Values represent the mean of 10 moving cells. Error bars were computed from scattering of values stacked in each column. N/S: non-stimulated (non-substrate) trophozoites were used as a negative control. The groups were analyzed using one-way Anova and Bonferroni posttest. ^***^P < 0.0001.

The non-stimulated trophozoites usually migrated poorly and without direction, which is clearly observed when superimposing the schemes of the cellular periphery of the representative trophozoite monitored every 20 s (Figures 1 A,B). Trophozoites monitored during the migration phase showed fast projections of pseudopods, pseudopods with filopodia, blebs, large rear uropod-shape body drag with filopodia behind the cell's body, tufts of filopodia, and occasionally lamellipodia. These trophozoites migrated persistently in the direction of the substrate (Figures 1 C,D). The velocity of migration of stimulated trophozoites was close to 1 μm/s (Figure 2) with no statistical differences between both chemoattractants, but significantly different with no-stimulated trophozoites.

**Figure 3:**
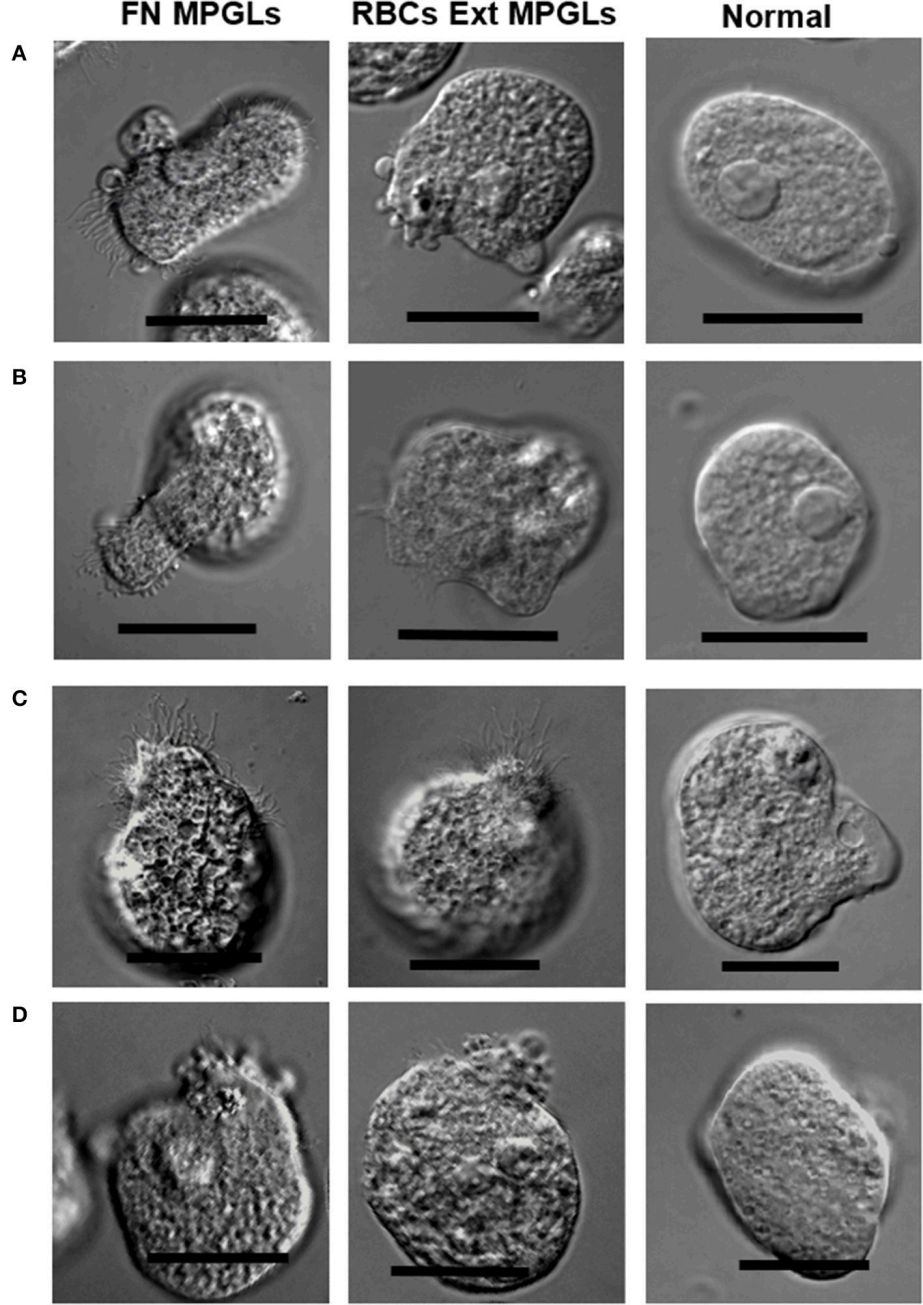
Membrane protrusions in migrating and non-migrating trophozoites. Trophozoites were incubated for 2 h at 37°C and then fixed. Images were obtained by light microscopy. FN MPGLs: trophozoites incubated on MPGLs using FN as substrate. RBCs MPGLs: trophozoites incubated on MPGLs using RBCs extracts as substrate. Normal: trophozoites incubated without stimulation. Scale bar: 20 μm.

Trophozoites that were monitored by light microscopy during adhesion to the MPGLs chambers generally showed short and large filopodia, blebs, and occasionally lamellipodia, which were not usually observed in non-stimulated trophozoites (Figure 3). Membrane projections of low thickness were difficult to observe by light microscopy (including filopodia), but better results were obtained by phase contrast using DIC microscopy. On the contrary, trophozoites that were carefully monitored during the transition from non-migration to migration phases frequently showed projecting pseudopods at the sites with the highest number of filopodia and were significantly different to normal cultured trophozoites (non-stimulated) when showing pseudopods (Figure 3C). To obtain long membrane protrusions, mainly large filopodia, it was necessary to maintain the temperature at 35–37°C during the process of observation of living cells as well as during fixation, because they showed to be sensitive to temperature variations and they retracted rapidly when the temperature was lowered.

**Figure 4:**
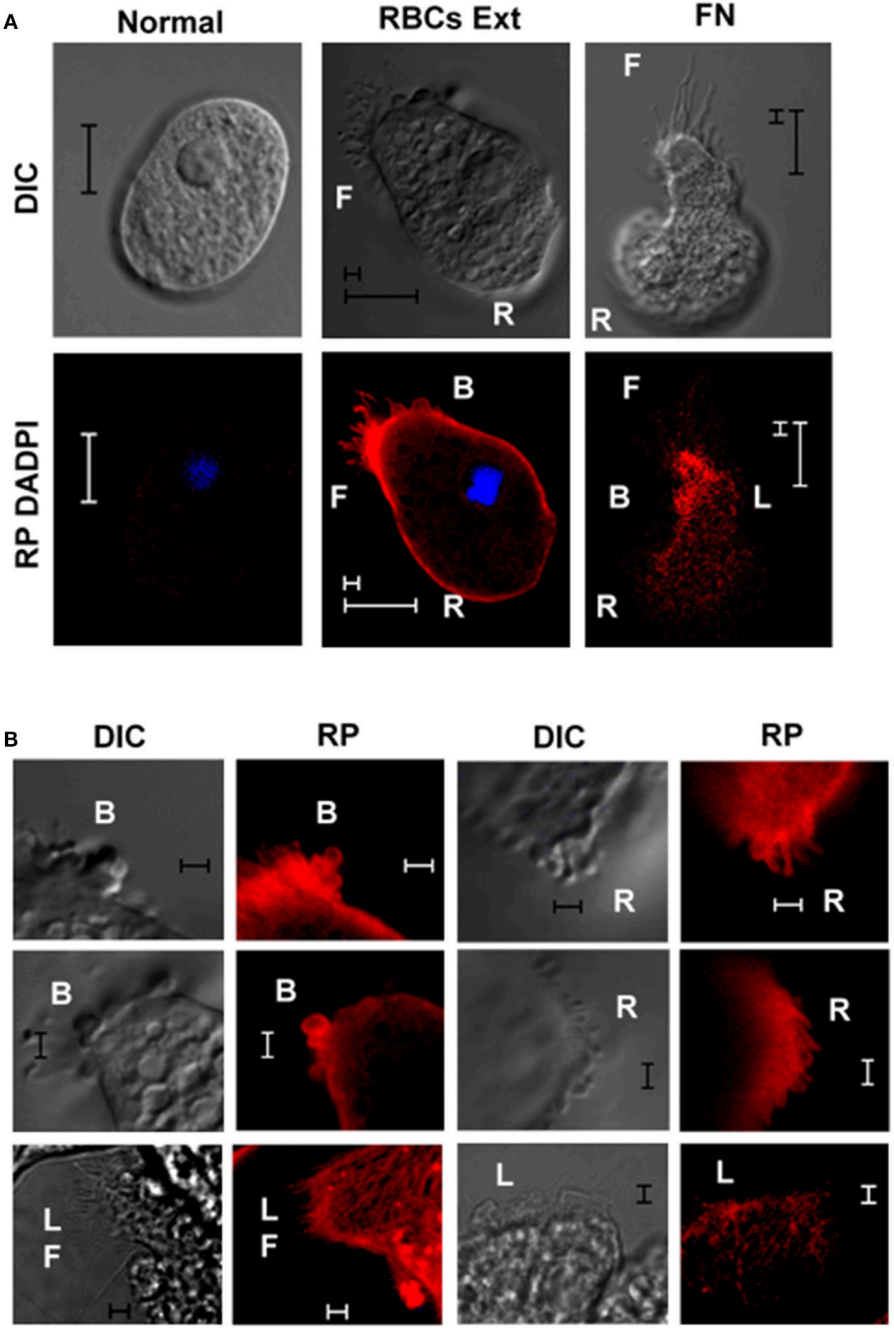
Distribution of F-actin in membrane protrusions promoted in MPGLs trophozoite migration. **(A)** Mobil trophozoites from MPGLs assay with erythrocyte extract or fibronectin as substrate were cultured for 120 min. Trophozoites from normal culture were used as negative control. **(B)** Representative protrusion showed by mobile trophozoites on MPGLs cultures. F-actin was detected using Rhodamine Phalloidin (RP). F: filopodia. R: retractile area. L: lamellipodia. B: blebs. Trophozoites were incubated on MPGLs for 120 min. Representative trophozoites of three independent experiments. **(A)** Scale bar: 10 μm and 2 μm. **(B)** Scale bar: 2 μm.

Because the actin filaments constitute the physical backbone of membrane protrusions in migrating cells, non-stimulated and stimulated trophozoites on the MPGLs were processed for the detection of F-actin. Short and long filopodia, large retractile uropods in the rear, blebs, and lamellipodia were stained for F-actin (Figure 4). However, short filopodia were entirely stained whereas long filopodia were partially stained (Figure 4A), and the actin in lamellipodia was less structured (Figure 4B) at the 120 min incubation. In some migrating trophozoites cortical actin was detected and commonly blebs were stained for F-actin.

**Figure 5:**
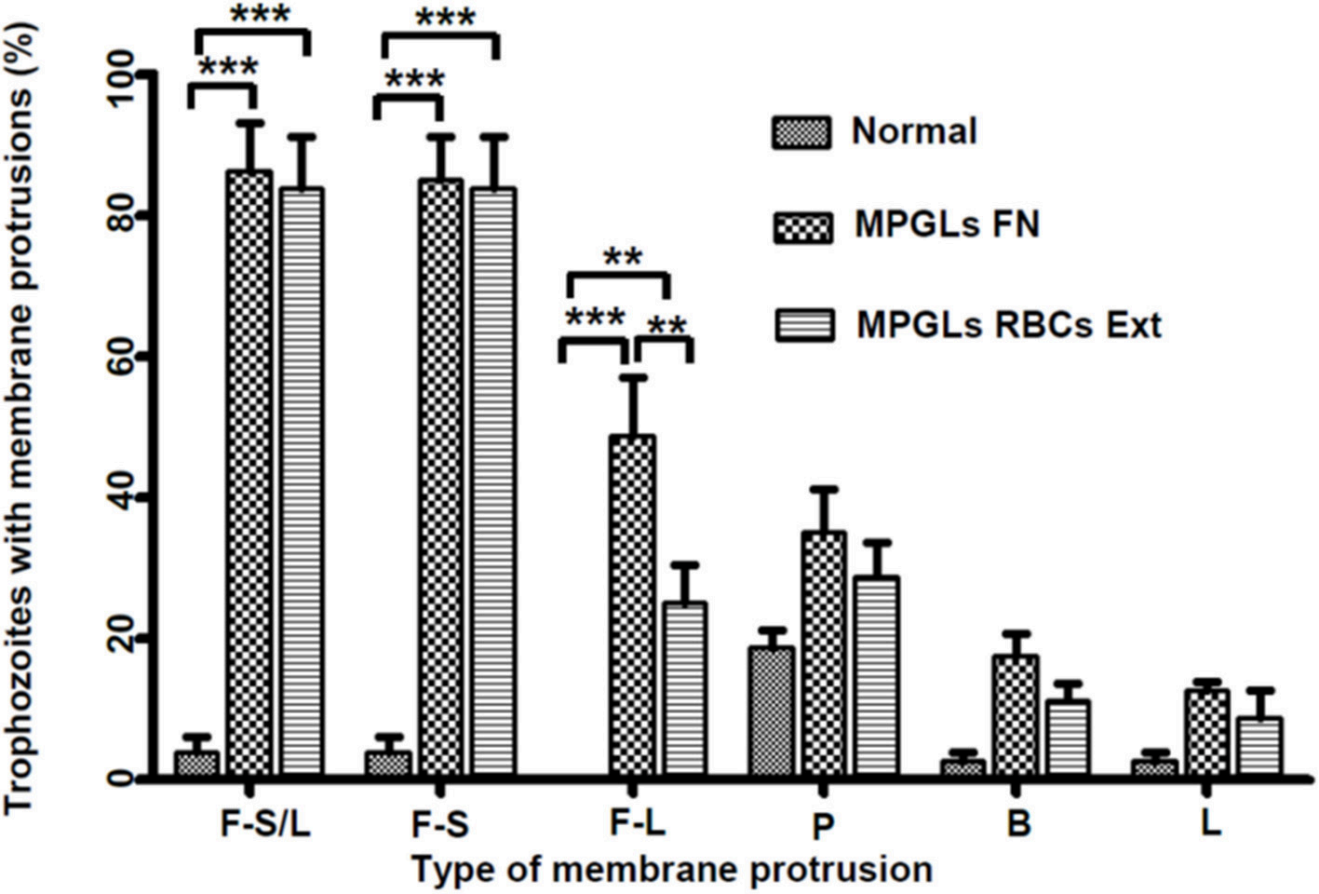
Membrane protrusions of mobile trophozoites. Cell membrane protrusion were determined in fixed cells after 120 min of culture on MPGLs (FN or RBCs extracts) or normal culture. The data represent the mean of protrusions observed in 80 cells for each group in four independent experiments. F-S/L, cells with short and long filopodia; F-S, cells with short filopodia; F-L, cells with long filopodia; P, cells with pseudopods; B, cells with blebs; L, cells with lamellipodia; The groups were analyzed using one-way Anova and Bonferroni posttest. ^***^P < 0.0001; ^**^P < 0.001.

With respect to the number of trophozoites that showed the different types of protrusions on the MPGLs, no statistically significant differences were found between the different types of protrusions induced by both components of the human host, except for the long filopodia, since a smaller number of trophozoites showed them when cultured on the erythrocyte extracts (Figure 5).

### Study of trophozoites incubated on MPGLs by SEM

**Figure 6:**
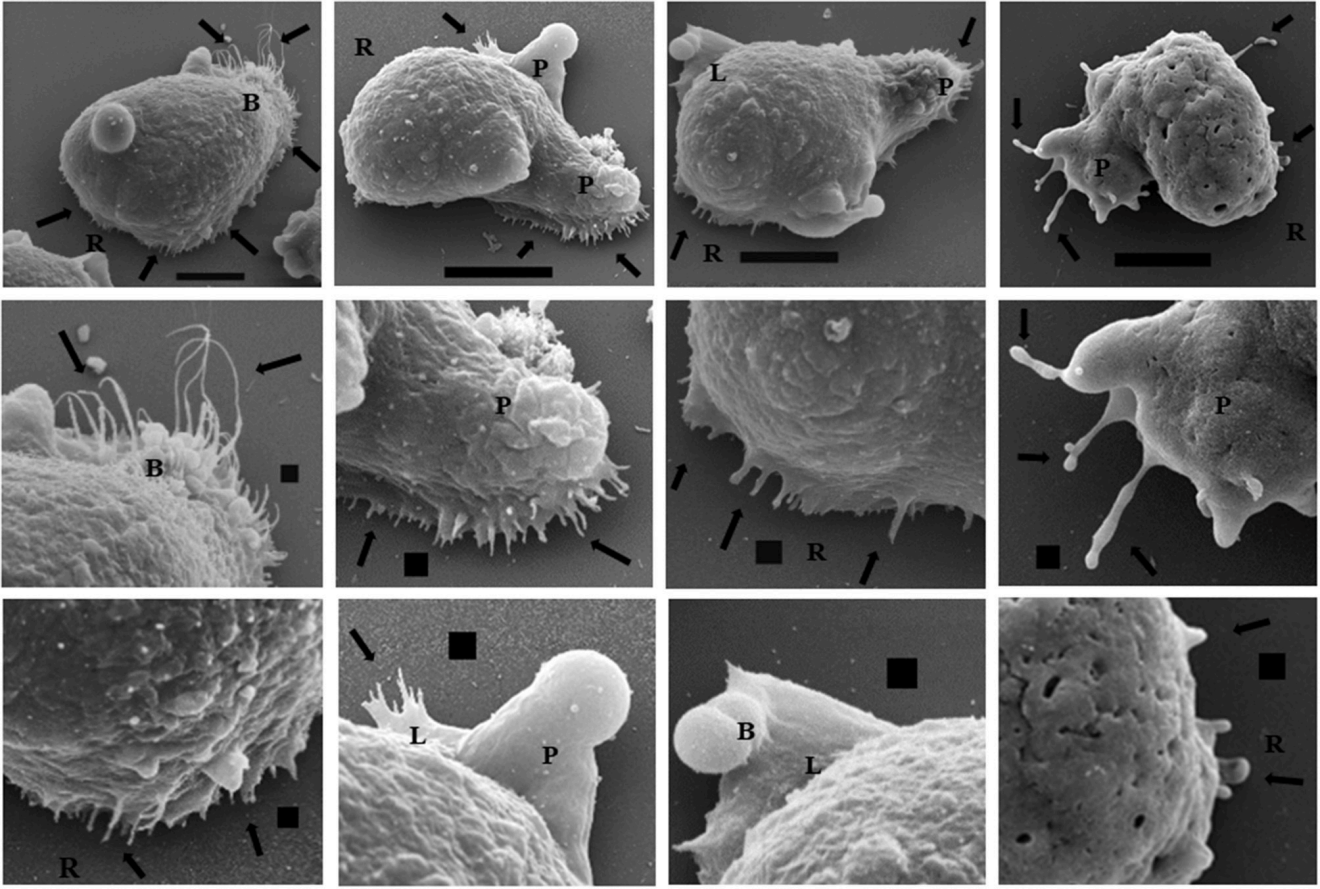
Scanning electron microscopy representative of membrane protuberances that eventually the trophozoites on MPGLs showed. Trophozoites were cultured on MPGLs for 120 min. (2-3) Maximization of the respective image (1) above. (A–D) Representative trophozoites morphology. Abundant long, thin, short and motile filopodia are marked with black arrows. L: lamellipodia. R: rear retractile zone. P: pseudopodia. B: blebs. Scale bar: 10 μm (1) and 1 μm (2, 3). Micrographs from right to left (A–D) respectively, and from top to bottom (1–3) respectively.

Trophozoites cultured on the MPGLs and analyzed by SEM showed pleomorphic cell morphology because they did not migrate in a synchronized manner. During MPGLs-trophozoites interaction (at 120 min), the trophozoites that moved on the MPGLs showed one or more combinations of the following structures: abundant filopodia (long, short, thin, and wide), pseudopods, lamellipodia, blebs, and a rear retractile zone (uroid with filopodia). Figure 6 shows representative images of some of the combinations of structures such as lamellipodia with filopodia (B3, B4), lamellipodia with blebs (B4), pseudopods with abundant filopodia (B1-2, C1), pseudopods with irregular filopodia (D1-D2), and structures at the retractile rear. Low-mobility trophozoites showed abundant filopodia (Figure 6A).

## Discussion

We set out to develop a method based on a substrate forming micropatterns on surfaces of glass or plastic and on *E. histolytica* trophozoite stimulation to produce a great variety of structures and membrane protrusions, including those that are generally observed with very low frequency. Micropatterning allows the control of cell adhesion on a surface with a substrate and proved to be a technique useful to answer several questions of cell biology (Scarpa et al., 2013), such as expression, polarization and compartmentalization of proteins; furthermore, cells internal organization (Lee et al., 2006; Théry et al., 2006), and control of cell-cell architecture (Bornens, 2008).

ECM components have been used to coat surfaces where trophozoites are grown, for example collagen and FN (Ramírez-Tapia et al., 2015; Talamás-Lara et al., 2015), and these interaction trophozoite-substrate leads to a response as the differential expression of proteins, protease secretion (Piña-Vázquez et al., 2012), and cytoskeletal rearrangements in which actin polymerization is actively involved (Sengupta et al., 2009; Javier-Reyna et al., 2012; Starke et al., 2014; Aguilar-Rojas et al., 2016). EMC components (such as FN) have been used to generate micropatterns in order to stimulate eukaryotic cells to migrate, produce abundant and different membrane protrusions (Alamdari et al., 2013; Starke et al., 2014). Based on this, we decided to use FN in discontinuous micropatterns in line to form what we denominated micropattern grill line (MPGL). We decided to use RBCs extracts in MPGL because *E. histolytica* trophozoites have been also shown to respond when in contact with erythrocytes and their residues (López-Revilla and Cano-Mancera, 1982; Boettner et al., 2005; Zaki et al., 2006). In recent years it has been known that cell protrusions and migration efficiency are dependent of the substrate geometry and the dose of the substrate (Starke et al., 2014) As a result we have obtained an optimal range of stimulation in our discontinuous design of micropatterns (MPGLs).

When moving, *E. histolytica* trophozoites usually show fast active pseudopodium protrusions and blebs in the front of the cell (Maugis et al., 2010), and occasionally retracting uropods at the rear (González-Robles and Martínez-Palomo, 1983; Marquay Markiewicz et al., 2011). Short and large filopodia are rarely produced, but filopodia of 1-6 micrometers extending between the trophozoites and MDKC or Caco-2 cell monolayers have been seen (Li et al., 1994), and in an “*ex-vivo* human intestinal model” (Bansal et al., 2009). In our design of MPGLs, when trophozoites were cultured for more than 1 h, the cells generated various membrane protrusions such as pseudopodia, lamellipodia, filopodia (including some larger than 10 μm), uropods and blebs. These protrusions were observed in trophozoites cultured either with FN or with RBCs as chemoattractants. Therefore, the MPGLs developed here could be a model quite useful for the study of these structures and for the search of proteins that participate in their function. In some cases the production of many trophozoite filopodia has been due to toxic lethal environmental conditions (Manna et al., 2013). In our case, the MPGLs did not cause the death of trophozoitesor alter their viability.

When making micropatterns on glass the components of these, such as ECM components, usually drop off of the surface after a day of cultivation (Alamdari et al., 2013); thus, for cultures designed to last only a few hours it was not necessary to stabilize them additionally, as in our MPGLs, where trophozoites were cultured less than 5 h. A wide variety of methods to make substrate micropatterns have been used as a tool to induce and analyze different cell types, which results in the induction of a variety of membrane protrusion in cancer cells (Théry, 2010; Paul et al., 2017; Tocco et al., 2018). Similarly, MPGLs were effective to increase significantly, a great amount and variety of trophozoites' membrane protrusions and to increase the motility in only a few hours of induction.

On the other hand, with the developed method it was possible to measure the velocity of migration with the chemoattractants used. This velocity was around 1 μm/s, which indicates that trophozoites are highly mobile cells compared to other cells analyzed in different systems. HT1080 fibrosarcoma cells displayed a mean velocity of 0.560–0.633 μm/min when plated in absence of FN to 0.695–0.761 μm/min on FN (Barry et al., 2015); *D. discoideum* seeded into a Dunn chemotaxis chamber with the external channel containing cAMP showed an average velocity of 5.9 μm/min (Nenasheva et al., 2012); NIH 3T3 cells presented a mean velocity of 2.96 μm/min on FN coated polyacrylamide gel and 4.23 μm/min on Collagen IV coated polyacrylamide gel (Pushkarsky et al., 2014); T24 cancer cells exhibited a velocity of 9.6 μm/h when cultured on Collagen gels (Laforgue et al., 2015); HT1080 fibrosarcoma cells presented an average migration velocity of 13.2 μm/h when visualized in vessels in live mice (Yamauchi et al., 2005).

Trophozoites, like other migratory cells (Yamaguchi and Condeelis, 2007; Jacquemet et al., 2015; Fritz-Laylin et al., 2017), showed filamentous actin in their membrane protrusions when stimulated in the MPGL, generating mainly long and short filipodia. The long filipodia did not show completely structured F-actin, possibly due to the fact that it was in the process of integration to stabilize the tubular filamentous structure (Karlsson et al., 2013).

Numerous membrane protrusions are produced in the trophozoites due to a migratory action that is promoted by some chemoattractants. Our results indicate that the way in which the substrate is imprinted can enhance the formation of structures such as abundant short and long filopodia since many trophozoites showed them when migrating over the MPGLs. Various cell types express high levels of long transient filopodia prior spreading (Partridge and Marcantonio, 2006), which suggests a highly conserved role of filopodia in mediating initial adhesion events and in exploring environmental features. In trophozoites, filopodia could be the sensory protrusion to detect chemoattractants to initiate directed migration.

Our experiments on MPGLs have also indicated that temperature of 36–37°C is crucial for the formation and maintenance of abundant filopodia because when the temperature was reduced to 30°C, the filopodia retracted (data not shown). During trophozoite migration assays on the MPGLs, two types of trophozoites were detected: those of rapid migration and those of slow or without migration. The last ones were found in the place where they were seeded and at a distance no greater than 3 mm from the chemoattractant. The highly mobile trophozoites showed the largest variety of membrane protrusions.

Trophozoites' motility plays an important role during invasive amoebiasis (Aguilar-Rojas et al., 2016). It has been proposed that both, physical forces and chemical signals are involved in the trophozoites' motility and migration (Leitch et al., 1988). However, the *in vivo* molecules that drive the chemotactic migration remain to be determined. We propose the MPGLs assay to study host's molecules that guide their chemotactic behavior, based on two considerations: this method has been shown to be reproducible, and the live image of cell movement and migration is quantifiable.

## Author contributions

FS-L designed, performed the experiments, analyzed results and participated in writing the manuscript. LB-P participated in the MPGLs assays. PE-G participated in experiments and was in charge of the *E. histolytica* cultures. AL-G participated in SEM experiments. BC-M participated in SEM experiments and the writing of the manuscript. JR-E, thesis director of Ph.D. student FS-L participated in the design, analysis and discussion of results, and in the writing of the manuscript.

### Conflict of interest statement

The authors declare that the research was conducted in the absence of any commercial or financial relationships that could be construed as a potential conflict of interest.

## Supplementary material

Supplementary Figure 1 Micropatterned Grill Lines. **(A)** Preparation of the MPGLs with FN or RBCs extracts at low and high concentrations, and observation of fresh dry MPGLs by light microscopy (40X, scale bar in μm). **(B)** Culture method on the MPGLs at 37°C by 1–5 h in TYI-S-33 medium. Cells were fixed with 4% p-formaldehyde or 2.5% (v/v) glutaraldehyde in 0.1 M sodium cacodylate buffer pH 7.2. **(C)** Culture of trophozoites on the MPGLs and light microscopy observation of the trophozoites' movements at 35–37°C.
