# Supplementary figures and images for: Influence of Micropatterned Grill Lines on Entamoeba histolytica Trophozoites Morphology and Migration
Source: Front Cell Infect Microbiol. 2018 Aug 24;8:295. doi: 10.3389/fcimb.2018.00295 (PMC6117912; doi:10.3389/fcimb.2018.00295)

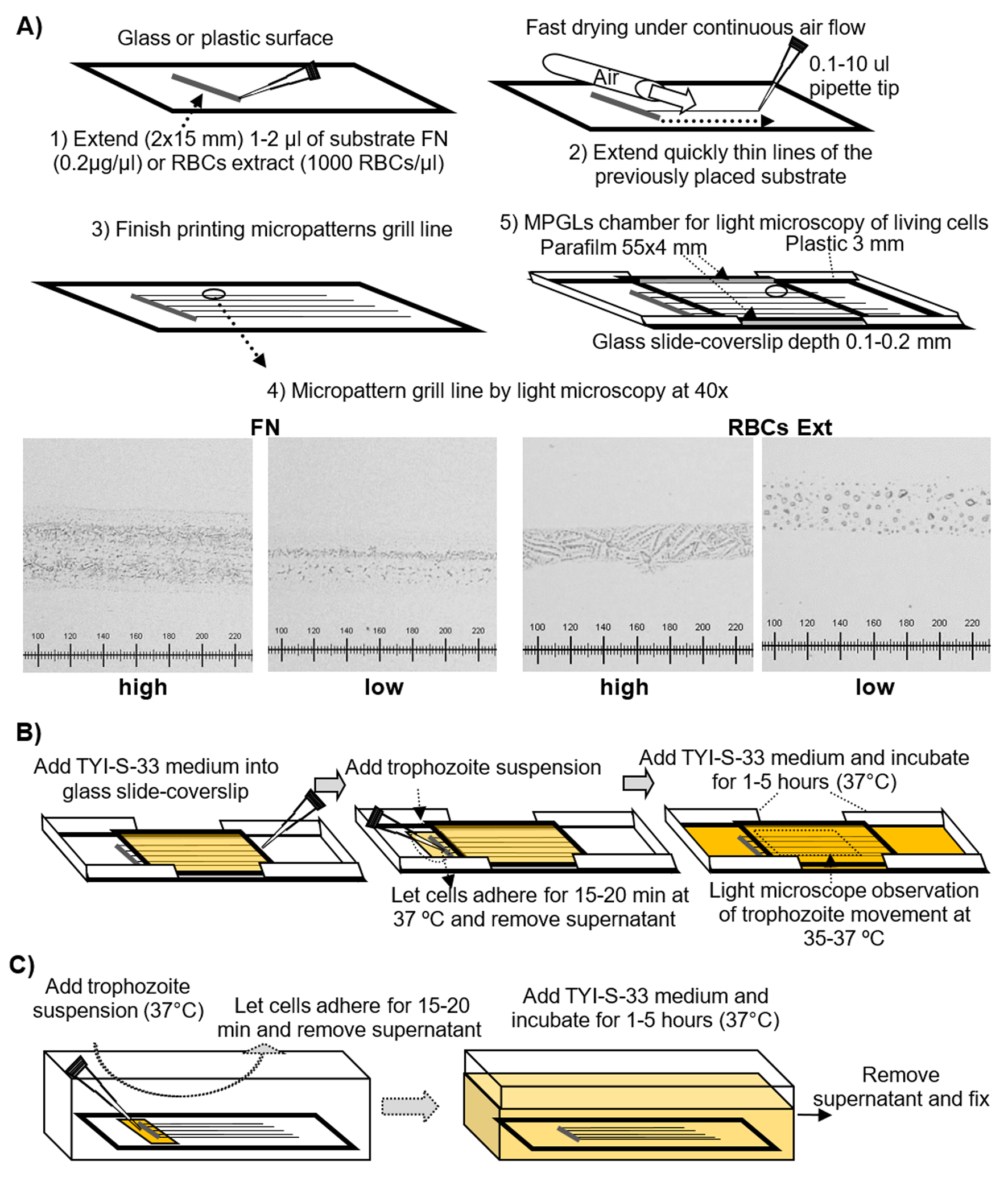

Supplement: Supplementary Figure 1 — Micropatterned Grill Lines. (A) Preparation of the MPGLs with FN or RBCs extracts at low and high concentrations, and observation of fresh dry MPGLs by light microscopy (40X, scale bar in μm). (B) Culture method on the MPGLs at 37°C by 1–5 h in TYI-S-33 medium. Cells were fixed with 4% p-formaldehyde or 2.5% (v/v) glutaraldehyde in 0.1 M sodium cacodylate buffer pH 7.2. (C) Culture of trophozoites on the MPGLs and light microscopy observation of the trophozoites' movements at 35–37°C. [file Image_1.TIF]
